# Regulatory mechanisms of luteolin in inflammatory respiratory diseases

**DOI:** 10.3389/fphar.2025.1720824

**Published:** 2025-11-27

**Authors:** Peidao Sun, Xiangchen Chen, Yuhang Wang, Xiaolin Wang, Kejing Li, Hongbo Song, Jinlong Mao

**Affiliations:** 1 The Affiliated Hospital, Shandong University of Traditional Chinese Medicine, Jinan, China; 2 College of Pharmacy, Shandong University of Traditional Chinese Medicine, Jinan, China; 3 College of Foreign Languages, Shandong University of Traditional Chinese Medicine, Jinan, China

**Keywords:** luteolin, pneumonia, acute lung injury, asthma, chronic obstructive pulmonary disease, pulmonary fibrosis, respiratory disease

## Abstract

Respiratory diseases continue to pose significant challenges to global public health, accounting for considerable morbidity and mortality. Medicinal plants have historically served as valuable sources of therapeutic agents, among which luteolin—a flavonoid abundant in various fruits, vegetables, and herbs—has recently garnered growing interest for its potential in treating inflammatory respiratory conditions. This review summarizes recent evidence on the pharmacological activities of luteolin in pneumonia, acute lung injury (ALI), asthma, chronic obstructive pulmonary disease (COPD), and pulmonary fibrosis. Data retrieved from PubMed, Web of Science, Google Scholar, CNKI, and Wanfang databases highlight that luteolin exerts multi-target protective effects through modulation of oxidative stress, inflammation, and immune responses. Specifically, luteolin suppresses NF-κB and MAPK signaling, activates the Nrf2/HO-1 pathway while inhibiting NOX4/NF-κB signaling, and downregulates TLR4/NF-κB signaling, NLRP3 inflammasome activation, and pyroptosis. In addition, it restores immune homeostasis by regulating macrophage polarization, balancing Th1/Th2 differentiation, and enhancing regulatory T cell (Treg) function. These results suggest that luteolin exhibits favorable safety and distribution profiles in the lung tissue in preclinical studies, highlighting its potential as a therapeutic candidate for inflammatory respiratory diseases. Nevertheless, further preclinical and clinical investigations are required to validate its efficacy, safety, and translational applicability in clinical practice.

## 1 Introduction

Respiratory diseases remain a leading cause of global disability and mortality, with their growing burden exacerbated by both traditional and emerging risk factors. While air pollution, smoking, and occupational exposures have long been recognized as contributors, global climate change and population aging have further accelerated the severity of these conditions (Deng et al., 2020; Liu et al., 2019). Chronic respiratory diseases, including COPD, asthma, and pneumoconiosis, rank as the third leading cause of death worldwide in 2019 (Momtazmanesh et al., 2023). ALI and acute respiratory distress syndrome (ARDS), which often arise from pneumonia, sepsis, or viral infections like SARS-CoV-2, present significant clinical challenges due to their rapid onset, high mortality, and limited therapeutic options (Matthay et al., 2024).

Current pharmacological treatments, such as glucocorticoids, leukotriene receptor antagonists, bronchodilators, and biologics, provide symptomatic relief but are limited by low target specificity, adverse effects, and potential drug resistance (Schleich et al., 2023). These limitations have spurred interest in bioactive natural products with multi-target potential. Flavonoids, including luteolin, quercetin, catechins, resveratrol, rutin, kaempferol, and hesperidin, have attracted attention due to their ability to modulate oxidative stress, inflammatory signaling, and immune responses (Hasnat et al., 2024; Xu et al., 2024).

**FIGURE 1:**
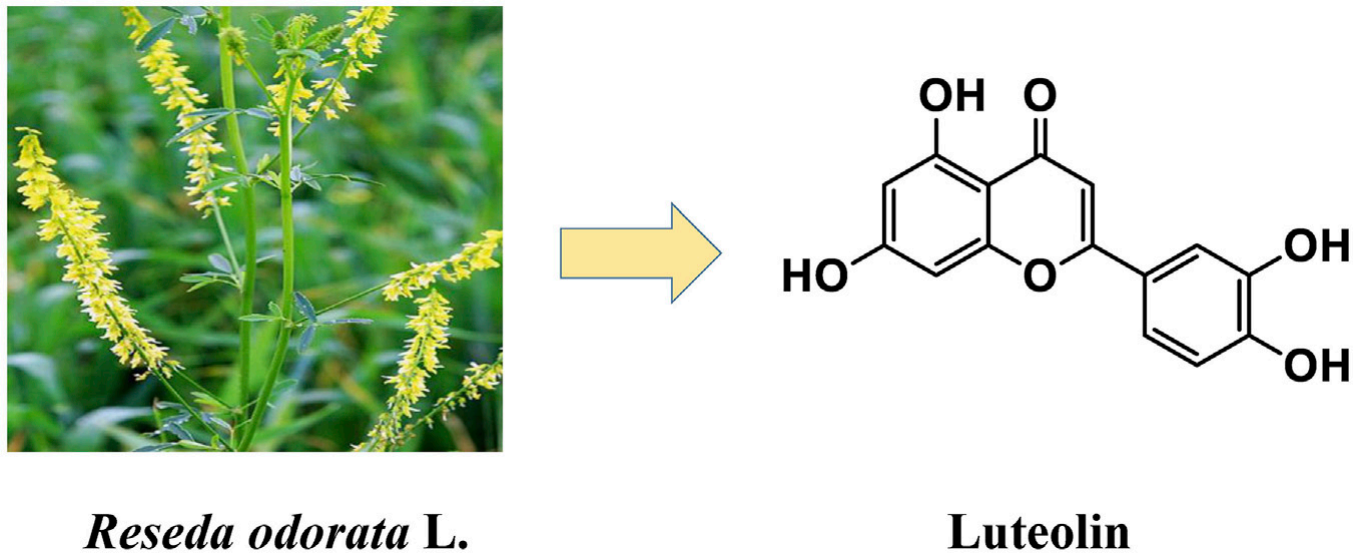
Chemical structure of luteolin originally identified from *Reseda odorata* (Resedaceae).

Luteolin, a naturally occurring flavone, was first identified from the branches, leaves, and stems of *Reseda odorata* L. (Resedaceae), as shown in Figure 1. It is widely distributed in fruits (e.g., apples, oranges), vegetables (e.g., oilseed rape, carrots, cucumbers, lettuce, and celery), as well as in peanut shells and corn silk (Zhang et al., 2024c; Zhu et al., 2024). In addition, luteolin is abundant in various medicinal herbs, including *Lonicera japonica* (honeysuckle) (Jia et al., 2023), *Plantaginis* Herba (Zhang et al., 2025), *Luffa acutangular* var. *amara* (Roxb.) (Kalaskar et al., 2025), *Taraxacum* (dandelion) (Liu Y. F. et al., 2024), *Brucea javanica* (He et al., 2020), *Buddleja officinalis* (Wei et al., 2022), *Schizonepeta tenuifolia* (Parhat et al., 2022), and *Salvia miltiorrhiza* (Wang L. et al., 2025).

Luteolin has been recognized for a wide range of biological effects, including antimicrobial, antiallergic, antitumor, neuroprotective, and cardioprotective properties, and shows therapeutic potential in diabetes, sepsis, and metabolic disorders (Hussain et al., 2023; Shi et al., 2024; Vajdi et al., 2023). However, despite extensive investigation of its pharmacological activities, systematic mechanistic reviews addressing its roles in respiratory diseases remain insufficient (Li et al., 2025; Lv et al., 2025; Zhang and Ma, 2024a).

In this review, we summarize current evidence on the molecular mechanisms of luteolin in inflammatory respiratory diseases, including pneumonia, ALI, asthma, COPD, and pulmonary fibrosis, with particular attention to its antioxidant, anti-inflammatory, and immunomodulatory actions. Overall, it highlights the therapeutic potential of luteolin and offers a mechanistic foundation for its future clinical investigations.

## 2 Methods—Literature search and selection strategy

A comprehensive literature search was performed to identify studies investigating the pharmacological and molecular effects of luteolin in respiratory diseases. Five electronic databases—PubMed, Web of Science, Google Scholar, China National Knowledge Infrastructure (CNKI), and Wanfang Data—were systematically searched from their inception to September 2025. Search strategies were adapted to the specific requirements of each database, using combined keywords and Medical Subject Headings (MeSH) terms. The English search terms included: “luteolin,” “pneumonia,” “acute lung injury,” “asthma,” “COPD,” “chronic obstructive pulmonary disease,” “pulmonary fibrosis,” “anti-inflammatory,” “antioxidant,” and “immune.” The Chinese terms comprised: “木犀草素” (luteolin), “肺炎” (pneumonia), “急性肺损伤” (acute lung injury), “哮喘” (asthma), “慢性阻塞性肺疾病” (COPD), and “肺纤维化” (pulmonary fibrosis). The search strategy was appropriately adjusted according to the characteristics of each database, with English and Chinese literature as the main references, and a total of 317 records were obtained through initial screening.

The inclusion criteria were defined as follows: study types included *in vitro* experiments, *in vivo* animal experiments, and clinical studies; research objects focused on respiratory disease models such as pneumonia, acute lung injury, asthma, COPD, and pulmonary fibrosis; intervention measures involved the therapeutic effects of luteolin (either as a pure compound or the main component of plant extracts); research content must address the effects of luteolin on oxidative stress, inflammatory response, immune regulation, or related signaling pathways (e.g., NF-κB, MAPK, Nrf2). The exclusion criteria included non-relevant studies (e.g., inflammation-related studies of luteolin in other diseases), reviews, comments, conference abstracts, or literature with inaccessible full texts; duplicate publications (for dissertations and published papers, only the one with the most complete data was retained); in terms of time range, priority was given to studies published in the past 5 years (2021–2025), while for diseases with few literature reports but with closely related experimental and clinical studies, the time restriction was relaxed.

The literature screening process was conducted as follows: after initial retrieval, duplicate records were eliminated first; preliminary screening was performed by reading titles and abstracts, and 218 potentially relevant records remained after applying the inclusion and exclusion criteria; full texts of these records were further obtained and read in detail, and those that did not meet the standards were excluded, resulting in 108 records finally included. Among the included records, 73 were experimental studies (*in vivo* and *in vitro*), accounting for 67.6%; four were clinically relevant studies, accounting for 3.7%; seven were pharmacokinetic studies, accounting for 6.5%; and 15 were reviews, accounting for 13.9%. Furthermore, we supplemented several recent research findings on other flavonoids to support and reinforce the therapeutic potential of luteolin in inflammatory respiratory diseases.

## 3 Mechanistic insights of luteolin in inflammatory respiratory diseases

Excessive inflammation represents a key pathological feature across multiple respiratory diseases, including pneumonia, asthma, COPD, ALI/ARDS, and pulmonary fibrosis. This inflammatory response is driven by cytokine release, immune cell infiltration, and dysregulated macrophage polarization. In infectious pneumonia, pathogens trigger robust cytokine release; COPD and asthma involve persistent, low-grade inflammation; ALI/ARDS are characterized by acute immune cell influx and cytokine storms; while pulmonary fibrosis is defined by progressive fibrotic remodeling with collagen deposition (Lambrecht and Hammad, 2015; Matthay et al., 2019). The imbalance in macrophage polarization, particularly the shift from anti-inflammatory M2 to pro-inflammatory M1 macrophages, further sustains chronic inflammation and can lead to irreversible tissue injury (Wynn and Vannella, 2016).

Luteolin exhibits multi-faceted activity against these processes, targeting oxidative stress, inflammatory signaling, and immune dysregulation. It directly scavenges reactive oxygen species (ROS) and chelates transition metal ions such as Fe^2+^ and Cu^2+^. Furthermore, luteolin activates the nuclear factor erythroid 2–related factor 2/heme oxygenase 1 (Nrf2/HO-1) pathway, upregulating key antioxidant enzymes including superoxide dismutase (SOD), catalase (CAT), glutathione (GSH), glutathione peroxidase (GPx), and elevating glutathione levels (Xia et al., 2024). The flavonoid also suppresses several pro-inflammatory signaling cascades such as nuclear factor kappa B (NF-κB), mitogen-activated protein kinase (MAPK), extracellular signal-regulated kinase (ERK), c-Jun N-terminal kinase (JNK), and phosphoinositide 3-kinase/protein kinase B (PI3K/Akt), resulting in decreased expression of pro-inflammatory mediators such as inducible nitric oxide synthase (iNOS), cyclooxygenase-2 (COX-2), interleukin-6 (IL-6), and tumor necrosis factor-α (TNF-α) (Aziz et al., 2018; Mahdiani et al., 2022). Beyond these effects, luteolin appears to promote immune homeostasis by favoring M2 macrophage polarization, restoring Th1/Th2 balance, and enhancing regulatory T cell (Treg) function and IL-10 expression (Hussain et al., 2023; Wynn and Vannella, 2016).

Preclinical studies provide consistent evidence supporting the efficacy of luteolin in mitigating respiratory inflammation. In a murine model of lipopolysaccharide (LPS)-induced ALI, luteolin attenuated pulmonary edema and neutrophil infiltration. This protective effect was attributed to the suppression of NF-κB and MAPK pathways, accompanied by reduced levels of IL-1β, IL-6, and TNF-α. Simultaneously, luteolin decreased lipid peroxidation (e.g., MDA) and enhanced the activity of key antioxidant enzymes including SOD, CAT, and GSH (Liu Z. et al., 2024). In ovalbumin (OVA)-induced asthmatic models, luteolin effectively alleviated airway inflammation, eosinophil infiltration, mucus hypersecretion, and airway hyperresponsiveness (AHR) through modulation of NF-κB signaling (Quan et al., 2024). More recently, in bleomycin-induced pulmonary fibrosis, hyaluronidase-functionalized luteolin nanoparticles (Lut@HAase) enabled targeted lung delivery, reduced collagen deposition and transforming growth factor-β1 (TGF-β1) expression, suppressed inflammation, improved lung function, and prolonged survival (Pan et al., 2024).

In summary, the available evidence suggests that luteolin acts as a multifaceted modulator of oxidative stress, inflammatory signaling, and immune homeostasis, supporting its therapeutic promise in inflammatory respiratory diseases.

## 4 Mechanisms of luteolin in respiratory diseases

### 4.1 Role of luteolin in pulmonary inflammation

Pneumonia involves alveolar and interstitial inflammation, typically triggered by bacterial, viral, or fungal pathogens. These pathogens activate immune and epithelial cells via pattern recognition receptors, resulting in the release of pro-inflammatory cytokines such as TNF-α and IL-6. Such responses promote neutrophil infiltration and alveolar damage, as summarized in Table 1.

**TABLE 1:** Potential molecular mechanisms of Luteolin in treating pneumonia, ALI and ARDS.

Disease	Type	Model	Dose	Duration	Effect	Mechanism	References
Antioxidant	Male SD rats	HPH	25, 50, 100 mg/kg, i.g	4 weeks	↓MDA,↑SOD, GPx, GSH; ↓HIF-1α	Antioxidant	Zhang et al. (2024b)
Antioxidant	Chinese hamster lung fibroblasts (V79-4)	H_2_O_2_	9 μmol/L	24 h	↑SOD, CAT, GPx; ↑HO-1	Antioxidant	Fernando et al. (2024)
Pneumonia	Female BALB/c mice	RSV	30, 60 mg/kg, i.g	3 days	↓ROS,↓MDA,↑SOD; ↓TNF-α, IL-1β, IL-6 mRNA; ↑IFN-β; ↓RSV	Antioxidant, glucose metabolism	Li et al. (2024a)
Inflammation	Mouse alveolar macrophage (MH-S) and peripheral macrophage (RAW 264.7)	LPS	25 μmol/L	24 h	↓ROS; ↓NO, PGE2; ↓TNF-α, IL-6; ↓iNOS, COX-2; ↑IL-1RA; ↓NF-κB; ↓AP-1	NF-κB and AP-1	Chen et al. (2007)
Bronchopneumonia	Male ICR mice	LPS	5, 10, 20 mg/kg	6 h	↓IL-1β, IL-6, TNF-α; ↓miR-132	miR-132/NF-κB	Liu and Meng (2018)
Bronchopneumonia	Human bronchial epithelial cell line BEAS-2B	LPS	1, 10, 100 μmol/L	24 h	↓IL-1β, IL-6, TNF-α; ↓miR-132; ↓p-p65/p65, p-I kB a/I kB a	miR-132/NF-κB	Liu and Meng (2018)
Inflammation	RAW264.7 murine macrophages and lung epithelial cells (A549)	Imiquimod (R837)	5, 10, 20 μmol/L	12 h	↓IL-6, TNF-α, IL-6, IL⁃1β; ↓IFN- β, IP-10; ↓p-p65, ↑p-GSK3β; ↓NF-κB; ↑HO-1, Nrf2	Nrf2/HO-1, NF-κB	Tao et al. (2024)
Inflammation	Male C57BL/6 mice	*P. aeruginosa*	20, 60 mg/kg, i.v	24 h	↓Infiltration, MPO; ↓Edema, W/D ratio; ↓IL-1β, IL-6, TNF-α, MIP-2; ↑IL-10; ↓PI3K, AKT, ERG, I kBa, c-Jun, c-Fos, NF-kBp65, AP-1 phosphorylation; ↓M1(NOS, CD86, IL-1β), ↑M2(Ym1, CD206, Arg1)	NF-κB and AP-1, M1/M2	Gu and Pang (2025)
Inflammation	Bone marrow-derived macrophage	*P. aeruginosa*	20, 40, 80 μmol/L	1 h	↓IL-1β, IL-6, TNF-α, MIP-2; ↑IL-10; ↓PI3K, AKT, ERG, I kBa, c-Jun, c-Fos, NF-kBp65, AP-1 phosphorylation; ↓M1(NOS, CD86, IL-1β), ↑M2(Ym1, CD206, Arg1)	NF-κB and AP-1, M1/M2	Gu and Pang (2025)
Inflammation	Lung tissue microvascular endothelial cells	fMLP	1, 10, 100 μmol/L		↓PDE4,↑cAMP-PDE; ↓VCAM-1,↔ICAM-1	PDE4 and cAMP	Kong et al. (2019)
Pneumonia	Male SD rats	LPS	1, 5, 10 mg/kg, i.p	6 h	↓Leukocyte infiltration; ↓W/D ratio; ↓sVCAM-1, ↔sE-selectin	PDE4 and cAMP	Kong et al. (2019)
Inflammation	RAW264.7 murine macrophages and lung epithelial cells (A549)	K.pneumoniae	20 μmol/L	24 h	↓IL-1β, IL-6, IL-12, IL-18, TNF-α; ↓iNOS; ↓CCL2, CCL3, CCL5, CCL7	Anti-inflammatory	Miao et al. (2021)
Inflammation	C57BL/6 mice	K.pneumoniae	0.5 mg/kg, i.v. (tail)	12 h	↓Infiltration; ↓Pulmonary edema	Anti-inflammatory	Miao et al. (2021)
ALI	Male C57BL/6 mice	LPS	15 mg/kg, i.g	24 h	↓Neutrophil infiltration; ↓p-AKT/AKT	Anti-inflammatory	Wang et al. (2024a)
ALI	Female albino Wistar rats	CLP	4 mg/kg, i.p	24 h	↓MDA,↑SOD, GSH; ↓TNF-α, IL-10; ↓Neutrophil infiltration,↓Septal thickening	Antioxidant,anti-inflammatory	Celebi et al. (2021)
ALI	RAW264.7 murine macrophages	LPS	20 μmol/L	2 h	↓IL-1β, IL-6, TNF-α; ↓TLR4, ↑IκBα, ↓NF-κB	TLR4/NF-κB	Mi et al. (2024)
ALI	Male SD rats	LPS	20, 40 mg/kg, i.g	3 days	↓ Infiltration, edema,W/D ratio; ↓MDA, ↑SOD; ↓IL-1β, IL-6, TNF-α; ↓TLR4, NF-κB p65	TLR4/NF-κB	Zou et al. (2021)
ALI	Female C57BL/6 mice	CLP	20, 40, 80 mg/kg, i.p	2 days	↓Neutrophil infiltration, MPO, edema, W/D ratio; ↓TNF-α, IL-6, IL-1β, MIP-2; ↓iNOS, COX-2; ↑HO-1; ↑IκBα, ↓NF-κB	NF-κB	Sun et al. (2019)
ALI	Male Swiss Albino mice	CLP	0.2 mg/kg, i.p	24 h	↑SOD, GSH; ↓IL-1β, IL-6, TNF-α; ↓ICAM-1, NF-κB, iNOS	NF-κB	Rungsung et al. (2018)
ALI	Male C57BL/6 mice	LPS	18, 35, 70 mg/kg, i.g	7 days	↓W/D ratio; ↓TNF-α, IL-6, IL-1β, IL-10	NF-κB	Jia et al. (2023)
ALI	Human alveolar epithelial cells (BEAS-2B)	LPS	10 μmol/L	24 h	↓TNF-α, IL-6, IL-1β, IL-10; ↓MyD88, ↓NF-κB p65, ↑IκBα; ↓Bax, ↑Bcl-2	NF-κB	Jia et al. (2023)
ALI	Male kunming mice	HgCl_2_	100 mg/kg, i.g	24 h	↓Neutrophils, MPO; ↓Edema, W/D ratio; ↓MDA, ↑SOD, GSH; ↑AKT/NRF2; ↑HO-1, NQO1; ↓IKK-α, ↑IκBα, ↓NF-κB; ↓TNF-α, IL-6, IL-1β	AKT/NRF2 and NF-κB	Liu et al. (2018)
ALI	RAW264.7 murine macrophages	LPS	10 μmol/L	24 h	↓TNF-α, IL-6; ↓NF-κB, ↓F-actin; ↓ROCK	F-actin and NF-κB	Cao (2021)
ALI	Male kunming mice	ECC	10 mg/kg, i.v. (tail)	2.5 h	↓Neutrophil infiltration; ↓Neutrophils, ↓Monocyte/macrophages; ↓TNF-α, IL-6	F-actin and NF-κB	Cao (2021)
ALI	Male ICR mice	LPS	5, 10, 20 mg/kg, i.p	6 h	↓MDA,↑SOD; ↓Neutrophil infiltration; ↓PMNs(CD45^+^/Ly6G^+^); ↓ TNF-α, KC, ↓ICAM-1; ↓p-ERK, p-p38 MAPK, p-JNK; ↑IκBα,↓NF-κB	MAPK and NF-κB	Kuo et al. (2011)
ALI	Neutrophils	fMLP/LPS	30 μmol/L	10 min	↓p-ERK, p-p38 MAPK, p-JNK; ↓p-Akt	MEK/ERK/Akt	Lee et al. (2010)
ALI	RAW264.7 macrophages	LPS	20 μmol/L	24 h	↑p-ERK1/2 (not JNK, p38 MAPK); ↑Ca^2+^ influx; ↑HO-1; ↓HMGB1, iNOS, COX-2; ↓NF-κB	ERK1/2 and NF-κB	Park et al. (2018)
ALI	RAW264.7 murine macrophages	LPS	3 μmol/L	24 h	↓ROS, NO; ↓TNF-α, IL-6, IL-1β; ↓p-p65, p-IκBα; ↓NF-κB; ↓p-p38, p-ERK; ↓MAPK	NF-κB and MAPK	Liu et al. (2024b)
ALI	Male BALB/c mice	LPS	15 mg/kg, i.p	10 days	↓Neutrophils, MPO; ↓Edema, W/D ratio; ↓MDA, ↑GSH; ↓TNF-α, IL-6; ↓p-p65, p-IκBα; ↓NF-κB; ↓p-p38, p-ERK; ↓MAPK	NF-κB and MAPK	Liu et al. (2024b)
Inflammation	RAW264.7 murine macrophages	LPS/IFN-γ, IL-4	20 μmol/L	24 h	↓p-STAT3, ↑p-STAT6; ↓M1(CD86, iNOS, IL-1β, IL-6), ↓IL-6, TNF-α; ↑M2(Arg1, CD206, CD163, IL-10, IL-13), ↑IL-10	M1/M2	Wang et al. (2020)
ALI	Male C57BL/6 mice	CLP	20 mg/kg, i.p	24 h	↓Neutrophils, MPO, ↓Edema, W/D ratio; ↓ROS, ↑GPx4; ↓IL-1β, IL-6, TNF-α, IL-17A; ↑Treg, FOXP3, IL-10; ↓caspase-1/11, GSDMD; ↓IL-1α, IL-1β	Treg/IL-10, caspase-1/11	Zhang et al. (2021b)
ALI/ARDS	Male C57BL/6 mice	CLP	0.2 mg/kg, i.p	24 h	↓Neutrophil infiltration, MPO, ↓edema, W/D ratio; ↑Treg (CD4^+^CD25^+^FOXP3^+^); ↓M1 (iNOS+), ↑M2(CD206+); ↓ p-p65; ↓NF-κB; ↓IL-1β, IL-6, TNF-α, IL-17A	Tregs, M1/M2	Xie et al. (2021)
ALI	Male BALB/c mice	LPS	25, 50, 100 mg/kg, i.p	24 h	↓p-BTK, p-FLT3; ↓CD6, CD4, CD19; ↓IL-1β, IL-6, IL-17, TNF-α; ↑IL-10	BTK/FLT3	Cao et al. (2025)
ALI	C57BL/6 mice	CLP	80 mg/kg, i.g	3 days	↓Infiltration, edema; ↓ROS, MDA; ↑SOD, CAT; ↓IL-6, TNF-α; ↓TXNIP, NLRP3, ASC, Caspase-1	ROS/TXNIP/NLRP3	Weng et al. (2023)
ARDS	Male BALB/c mice	LPS	20 mg/kg, i.p	5 days	↓Infiltration, ↓Edema, W/D ratio, ↑ PaO_2_; ↓ROS, ↑GPx, CAT; ↓IL-18, IL-1β, TNF-α; ↓TXNIP, NLRP3, caspase-1, caspase-4	ROS/TXNIP/NLRP3	Qu et al. (2022)
ALI	C57BL/6J mouse pups	CSI	10 mg/kg, i.p	9 h	↓Infiltration; ↓Bax/Bcl2; HIF-1a, NLRP3	HIF-1a and NLRP3	Zhang et al. (2021a)
Pyroptosis	Mouse mononuclear macrophage (J774A.1)	LPS + ATP	2, 4, 8 μmol/L	12 h	↓PI^+^ cells, LDH release; ↓IL-1β, IL-18; ↓NLRP3, ASC, Caspase-1, GSDMD	NLRP3/Caspase-1/GSDMD	Liu et al. (2025)
Pyroptosis	Male ICR mice	LPS	40, 60, 80 mg/kg, i.p	3 days	↓IL-1β, IL-18; ↓NLRP3, ASC, Caspase-1, GSDMD	NLRP3/Caspase-1/GSDMD	Liu et al. (2025)
ALI/ARDS	Male BALB/c mice	LPS	40 mg/kg, i.p	24 h	↑AFC; ↓infiltration, W/D ratio; ↑cGMP; ↑α/γ-ENaC	ENaC	Hou et al. (2022)
ALI	Male BALB/c mice	LPS	20 mg/kg, i.p	24 h	↑Alveolar structure; ↓Inflammatory infiltration; ↑cGMP; ↑α/γ-ENaC	ENaC	Liu et al. (2020)
ALI	Male BALB/c mice	LPS	40 mg/kg, i.p	24 h	↓Infiltration, edema, W/D ratio; ↓p-JAK2, p-STAT3, ↑SOCS3; ↑ENaC	ENaC	Chen et al. (2023)

↑ = upregulated; ↓ = downregulated.

#### 4.1.1 Attenuation of oxidative stress and mitochondrial injury

Oxidative stress plays a key role in various forms of lung injury, exacerbating mitochondrial impairment, immune imbalance, and epithelial damage. In a rat model of hypoxic pulmonary hypertension (HPH), luteolin (50 mg/kg, i.g.) was shown to alleviate oxidative damage by directly scavenging ROS, including superoxide anions (O_2_^−^) and hydroxyl radicals (·OH), while reducing malondialdehyde (MDA) levels and restoring endogenous antioxidants, such as the activities of SOD and GPx, and the levels of GSH. Moreover, luteolin preserved mitochondrial integrity by preventing swelling, cristae disruption, and fragmentation, while concurrently downregulating HIF-1α expression, thereby supporting mitochondrial respiration and mitigating injury signaling (Zhang et al., 2024b).

In pulmonary fibroblasts (V79-4), luteolin (2.5 mg/L) suppressed hydrogen peroxide (H_2_O_2_)–induced lipid peroxidation and caspase-3/9 activation, while enhancing the activity of antioxidant enzymes (SOD, CAT, GPx) and upregulating HO-1 expression, thereby attenuating oxidative stress–induced apoptosis (Fernando et al., 2024). Similarly, in a murine model infected with respiratory syncytial virus (RSV), luteolin-7-O-glucoside (LUT-7G; 60 mg/kg, i.g.) lowered ROS and MDA levels, preserved SOD activity, and stabilized mitochondrial membrane potential (ΔΨm). This was accompanied by reduced TNF-α, IL-1β, and IL-6 levels, increased interferon-β (IFN-β) expression, suppressed viral replication, and restored pulmonary immunometabolic homeostasis (Li J. et al., 2024).

#### 4.1.2 Inflammatory signaling and immune regulation

Luteolin modulates multiple inflammatory signaling cascades, including NF-κB, activator protein-1 (AP-1), and PI3K/Akt. In LPS-stimulated MH-S and RAW264.7 macrophages, luteolin (25 μmol/L) inhibited NF-κB and AP-1 activation, leading to decreased release of prostaglandin E2 (PGE2), TNF-α, and IL-6 (Chen et al., 2007). Consistently, in LPS-induced murine models, luteolin (18–70 mg/kg) downregulated microRNA-132 (miR-132), suppressed NF-κB signaling, and reduced IL-1β, IL-6, and TNF-α expression, thereby alleviating airway inflammation (Liu and Meng, 2018). In a viral mimic model of influenza-infected RAW264.7 macrophages, luteolin (20 μmol/L) not only lowered IL-6, IL-1β, TNF-α, IFN-β, and interferon-inducible protein 10 (IP-10) levels but also activated the Nrf2/HO-1 axis while suppressing NF-κB via reduced p65 phosphorylation and increased phosphorylation of glycogen synthase kinase-3β (GSK3β) (Tao et al., 2024). This dual regulation suggests a coordinated anti-inflammatory and immunomodulatory mechanism.

In a murine model of acute *Pseudomonas aeruginosa* pulmonary infection, luteolin (60 mg/kg, i.v.) inhibited epidermal growth factor receptor (EGFR) phosphorylation, thereby attenuating downstream PI3K/Akt/NF-κB and ERK/AP-1 signaling (Gu and Pang, 2025). Luteolin also modulated macrophage polarization by downregulating M1-associated markers (iNOS, CD86, IL-1β) and upregulating M2 markers (Arg1, Ym1, CD206), accompanied by elevated IL-10 levels, thereby promoting a reparative pulmonary microenvironment (Gu and Pang, 2025).

Luteolin has demonstrated vascular-protective effects in endothelial dysfunction models by increasing cyclic adenosine monophosphate (cAMP) through the inhibition of phosphodiesterase (PDEs), particularly PDE4. In pulmonary microvascular endothelial cells, luteolin (100 μmol/L) selectively reduced vascular cell adhesion molecule-1 (VCAM-1) expression without affecting intercellular adhesion molecule-1 (ICAM-1) (Kong et al., 2019). Furthermore, in an LPS-induced pneumonia model, luteolin (10 mg/kg, i.p.) significantly reduced the levels of soluble ICAM-1 (sICAM-1) but had no significant effect on soluble E-selectin (sE-selectin). *In vivo* studies further confirmed that luteolin effectively alleviated leukocyte infiltration and pulmonary edema by attenuating vascular inflammation through PDE4-mediated cAMP elevation (Kong et al., 2019).

Recent evidence indicates that wind-chill exposure triggers pulmonary inflammation through the TRPM8–TLR4–NF-κB axis, underscoring temperature shifts as significant external inflammatory stimuli (Wu et al., 2025a). This mechanism supports the possibility that luteolin’s anti-inflammatory effects extend to non-infectious insults, such as cold-induced lung damage.

#### 4.1.3 Inhibition of bacterial virulence and enhancement of host defense

Beyond host immune modulation, luteolin also exerts protective effects by targeting pathogen virulence factors. In pulmonary epithelial cells infected with *Klebsiella pneumoniae*, luteolin (20 μmol/L) was found to disrupt bacterial adhesion and enhance macrophage phagocytic and bactericidal functions, which coincided with reduced levels of IL-1β, IL-6, IL-12, and IL-18 (Miao et al., 2021). In corresponding murine models, luteolin (0.5 mg/kg, i.v.) reduced pulmonary bacterial load and inflammatory cell infiltration, thereby preserving alveolar integrity (Miao et al., 2021).

In *P. aeruginosa* models, luteolin (10–50 μmol/L) not only inhibited bacterial growth directly but also attenuated EGFR-mediated inflammatory signaling, leading to reduced pulmonary permeability and neutrophil infiltration (Gu and Pang, 2025). Moreover, in *Staphylococcus aureus* infection models, luteolin (100 mg/kg) disrupted the accessory gene regulator (agr) quorum-sensing system, thereby reducing bacterial virulence, pulmonary bacterial load, and tissue damage. The abolition of these protective effects in agr-deficient strains confirms the specificity of luteolin’s action through this quorum-sensing mechanism (Yuan et al., 2022).

### 4.2 Role of luteolin in lung injury

**FIGURE 2:**
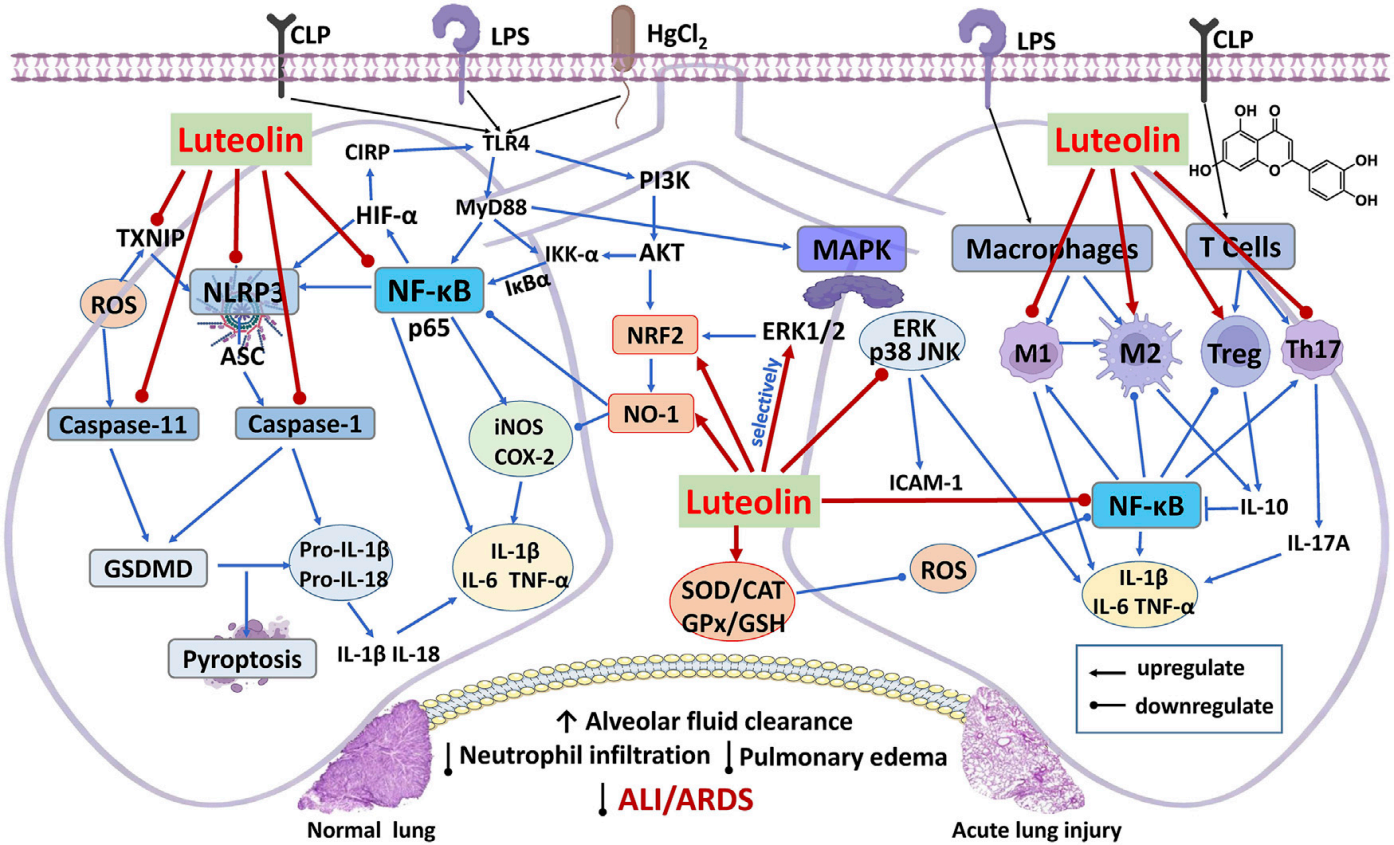
Network regulatory mechanisms of luteolin in treating ALI and ARDS.

Acute lung injury (ALI), caused by both infectious and non-infectious sources such as severe trauma, hyperoxia, and sepsis, is characterized by dysregulated inflammatory responses, including massive release of cytokines (e.g., TNF-α, IL-1β, IL-6, and IL-8). This inflammatory cascade disrupts the alveolar-capillary barrier and may progress to acute respiratory distress syndrome (ARDS). The potential protective mechanisms of luteolin are illustrated in Figure 2 and summarized in Table 1.

#### 4.2.1 Suppression of the NF-κB signaling pathway

Substantial evidence supports that luteolin alleviates ALI largely through inhibiting NF-κB activation. In LPS-induced murine models, luteolin (15 mg/kg, i.g.) reduced alveolar inflammatory cell infiltration and alveolar wall thickening, and improved lung tissue architecture by inhibiting AKT phosphorylation (Wang X. F. et al., 2024). Similar protective effects were observed in cecal ligation and puncture (CLP)-induced septic rats, where luteolin (4 mg/kg, i.p.) reduced MDA levels, increased SOD and GSH activities, and decreased TNF-α levels (Celebi et al., 2021). Further studies confirmed that luteolin inhibits Toll-like receptor 4 (TLR4)/NF-κB signaling and downstream pro-inflammatory cytokines (IL-1β, IL-6, TNF-α) in LPS-induced ALI models (Mi et al., 2024; Zou et al., 2021). In CLP-induced ALI models, luteolin (20 mg/kg, i.g.) also increased HO-1 expression while reducing the expression of iNOS, COX-2, and ICAM-1 in septic mice (Rungsung et al., 2018; Sun et al., 2019). Notably, luteolin-rich extracts from *L. japonica* also exert protective effects through the NF-κB pathway (Jia et al., 2023).

In a mercuric chloride (HgCl_2_)-induced model, luteolin (100 mg/kg, i.g.) markedly reduced neutrophil infiltration and pulmonary edema, as indicated by a decreased lung wet-to-dry (W/D) ratio. These protective effects are associated with activation of the AKT/NRF2 pathway, upregulation of antioxidant enzymes including HO-1 and NAD(P)H quinone dehydrogenase 1 (NQO1), and concurrent NF-κB inhibition, as evidenced by reduced IκB kinase alpha (IKK-α) activation, decreased degradation of Inhibitor of kappa B alpha (IκBα), and suppressed NF-κB nuclear translocation, ultimately resulting in decreased levels of TNF-α, IL-6, and IL-1β (Liu et al., 2018). *In vitro*, luteolin (10 μmol/L) decreased TNF-α and IL-6 levels in RAW264.7 macrophages by interfering with Rho/ROCK signaling and F-actin reorganization, thereby suppressing NF-κB activity and alleviating extracorporeal circulation (ECC)–induced inflammatory responses (Cao, 2021).

Recent evidence shows that SIRT6 activation, either through adenoviral overexpression or small-molecule stimulation by UBCS039, suppresses LPS-induced VCAM-1/ICAM-1 expression and NF-κB activity in human lung microvascular endothelial cells (HLMVECs), thereby alleviating pulmonary microvascular inflammation and suggesting therapeutic potential for lung injury (Wang J. et al., 2024). Considering that luteolin also inhibits NF-κB signaling in ALI models, it may partially exert its protective effects through SIRT6-mediated mechanisms.

#### 4.2.2 Inhibition of the MAPK signaling pathway

The MAPK family—including p38, ERK, and JNK—contributes to ALI progression by regulating neutrophil recruitment, oxidative stress, and pro-inflammatory cytokine release. In LPS-induced murine models, luteolin (20 mg/kg, i.p.) markedly reduced vascular permeability and neutrophil infiltration, lowered MDA levels while restoring SOD activity. These changes were accompanied by inhibition of p38, ERK, and JNK phosphorylation, along with downregulation of TNF-α, keratinocyte chemoattractant (KC), and ICAM-1 (Kuo et al., 2011).

In activated neutrophil models stimulated with (formyl-Met-Leu-Phe) fMLP and LPS, luteolin (30 μmol/L) suppressed neutrophil cell infiltration and respiratory burst, decreased phosphorylation of mitogen-activated protein kinase (MEK), ERK, and PI3K/Akt, confirming its anti-inflammatory effects through MAPK inhibition (Lee et al., 2010). Interestingly, under certain stress conditions, luteolin (20 μmol/L) was found to selectively activate ERK1/2 without affecting JNK or p38, and this ERK activation promoted HO-1 upregulation in a calcium-dependent manner, while concurrently suppressing high mobility group box 1 (HMGB1), iNOS, COX-2, and NF-κB expression, highlighting a context-dependent role for luteolin in MAPK modulation (Park et al., 2018).

Additionally, luteolin (15 mg/kg, i.p.), either alone or in combination with paeoniflorin (75 mg/kg, i.p.), synergistically inhibits MAPK (p-p38, p-ERK) and NF-κB (p-p65, p-IκBα) pathways (Liu Z. et al., 2024). Recent evidence indicates that luteolin also alleviates TNF-α-induced microvascular endothelial inflammation by concomitantly inhibiting the phosphorylation of MAPK, NF-κB, and AKT signaling. The anti-inflammatory efficacy is further enhanced by the synergistic suppression between the MAPK and NF-κB pathways (Lu et al., 2024).

#### 4.2.3 Regulation of immune cell polarization and function

Dysregulated immune responses—particularly imbalances in macrophage polarization and Treg function—play a critical role in acute lung injury (ALI). In macrophages polarized toward M1 (by LPS + IFN-γ) or M2 (by IL-4) phenotypes, luteolin (20 μmol/L) shifted the balance toward the M2 phenotype by suppressing M1-associated STAT3 phosphorylation while enhancing M2-promoting STAT6 phosphorylation. This shift corresponded with decreased expression of M1 markers (CD86, iNOS, IL-1β, IL-6) and inflammatory cytokines (IL-6, TNF-α), alongside increased M2 markers (Arg1, CD206, CD163, IL-10, IL-13) and elevated IL-10 levels (Wang et al., 2020). Similarly, cerium–luteolin nanocomposites promoted M2 polarization and reduced lung injury in LPS-induced mice (Gu et al., 2024).

Beyond innate immunity, luteolin also modulates adaptive immune responses by promoting the expansion of regulatory T cells (Tregs). In CLP-induced models, luteolin (20 mg/kg, i.p.) restored circulating and splenic Treg populations, increased pulmonary FOXP3 and IL-10 levels in both serum and bronchoalveolar lavage fluid (BALF), thereby alleviating lung injury in septic mice (Zhang Z. T. et al., 2021). At a lower dose (0.2 mg/kg, i.p.), luteolin expanded the proportion of CD4^+^CD25^+^FOXP3^+^ Tregs and upregulated FOXP3 expression, which may indirectly rebalanced macrophage polarization by suppressing M1 and promoting M2 phenotypes. These effects coincided with reduced NF-κB p65 phosphorylation, thereby decreasing the levels of IL-1β, IL-6, TNF-α, and IL-17A, ultimately alleviating pulmonary edema, neutrophil infiltration, and lung injury (Xie et al., 2021).

Recently, novel evidence has implicated luteolin in modulating tyrosine kinase–driven immune overactivation. In LPS-induced ALI, luteolin (100 mg/kg, i.p.) inhibited phosphorylation of Bruton’s tyrosine kinase (BTK) and Fms-like tyrosine kinase 3 (FLT3), limited infiltration of CD68^+^ macrophages, CD4^+^ T cells, and CD19^+^ B cells, and modulated cytokine levels by decreasing IL-1β, IL-6, IL-17, and TNF-α while increasing IL-10 (Cao et al., 2025). These results indicate that luteolin exerts broad immunomodulatory effects across both innate and adaptive immune responses to promote the resolution of inflammation in ALI models.

#### 4.2.4 Suppression of the NLRP3 inflammasome pathway

The NOD-like receptor pyrin domain–containing 3 (NLRP3) inflammasome serves as a central mediator of pulmonary inflammatory injury, typically activated by upstream signals such as ROS and thioredoxin-interacting protein (TXNIP). In both CLP- and LPS-induced ALI models, luteolin (80 mg/kg, i.g.) decreased ROS and MDA levels, restored endogenous antioxidants (SOD, CAT), and suppressed TXNIP, caspase-1, and NLRP3 expression. These changes resulted in reduced IL-1β and IL-18 release and inhibition of TXNIP/NLRP3 complex formation, thereby mitigating the ALI/ARDS pathology (Qu et al., 2022; Weng et al., 2023). Importantly, the protective effects of luteolin were absent in TXNIP-deficient models, confirming TXNIP as a specific upstream target (Wang X. et al., 2021).

Additionally, luteolin also downregulates cold-inducible RNA-binding protein (CIRP), an early inflammatory mediator in the lung. In neonatal sepsis models, it inhibited HIF-1α and NLRP3 in recruited macrophages, thereby reducing inflammatory cell infiltration and epithelial apoptosis (Zhang Y. et al., 2021). *In vitro*, luteolin (8 μmol/L) suppressed inflammasome complex assembly and gasdermin D (GSDMD) cleavage, preventing pyroptosis induced by LPS/adenosine triphosphate (ATP). Molecular docking analyses further support a model in which luteolin directly interacts with core inflammasome components, providing a structural basis for its inhibitory activity (Liu et al., 2025).

Recent evidence shows that the natural compound 5-deoxy-rutaecarpine directly inhibits NLRP3 inflammasome activation and alleviates inflammatory infiltration in LPS-induced ALI (Luo et al., 2025). This finding closely parallels luteolin’s ability to suppress NLRP3-driven pyroptosis, reinforcing the therapeutic potential of modulating the NLRP3 pathway in ALI/ARDS.

#### 4.2.5 Inhibition of caspase-11/GSDMD-mediated non-canonical pyroptosis

Besides canonical inflammasome pathways, luteolin also blocks the non-canonical pyroptosis pathway driven by caspase-11 and GSDMD. In CLP-induced ALI models, luteolin (20 mg/kg, i.p.) reduced inflammatory cytokines (IL-1β, IL-6, TNF-α, IL-17A) in serum and BALF, decreased neutrophil infiltration and myeloperoxidase (MPO) activity, and alleviated pulmonary edema, as indicated by decreased W/D ratios. It also ameliorated oxidative stress, marked by reduced ROS and elevated GPx4 activity. Simultaneously, luteolin suppressed the expression of caspase-1, caspase-11, and GSDMD, along with reduced IL-1α and IL-1β levels, indicating dual inhibition of both canonical and non-canonical pyroptosis (Zhang Z. T. et al., 2021; Zhang, 2022).

A recent study further showed that luteolin (20 mg/kg) inhibits the AKT1/nitric oxide synthase 2/cathepsin G (AKT1/NOS2/CTSG) axis, thereby blocking caspase-11 and GSDMD activation, leading to reduced IL-1β and TNF-α release, thus mitigating sepsis-induced pulmonary injury (Zhang et al., 2023).

#### 4.2.6 Modulation of ENaC in alveolar epithelium

The epithelial sodium channel (ENaC) plays a critical role in alveolar fluid clearance and alleviating pulmonary edema. Its activity is negatively regulated by cyclic guanosine monophosphate (cGMP), resulting in the inhibition of sodium reabsorption. In LPS-induced ALI, luteolin (20 mg/kg, i.p.) increased cGMP levels and activated cGMP/PI3K signaling. This resulted in the upregulation of α- and γ-ENaC subunits, enhanced alveolar fluid clearance, and reduced inflammation and pulmonary edema, collectively attenuating ALI/ARDS pathology (Hou et al., 2022; Liu et al., 2020).

In another study, luteolin (20 mg/kg, i.p.) inhibited the Janus kinase 2/signal transducer and activator of transcription 3 (JAK2/STAT3) phosphorylation and elevated suppressor of cytokine signaling 3 (SOCS3) expression, further sustaining ENaC activity, enhancing fluid resorption, and reducing inflammatory infiltration (Chen et al., 2023). Collectively, these data underscore the regulation of ENaC as a pivotal mechanism by which luteolin mitigates alveolar edema in ALI and ARDS.

### 4.3 Mechanisms of luteolin in asthma

**FIGURE 3:**
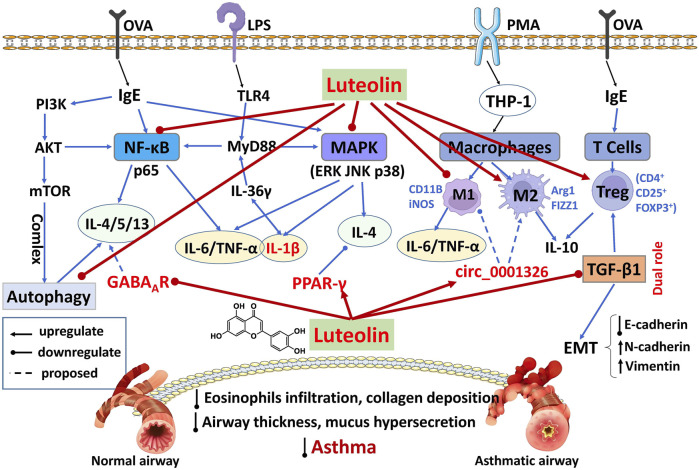
Network regulatory mechanisms of luteolin in treating asthma.

Allergic asthma is a heterogeneous inflammatory disorder driven largely by dysregulated NF-κB signaling, leading to airway hyperresponsiveness (AHR), chronic inflammation, and mucus overproduction. The potential protective mechanisms of luteolin are illustrated in Figure 3 and summarized in Table 2.

**TABLE 2:** Potential molecular mechanisms of Luteolin in treating asthma.

Disease	Type	Model	Dose	Duration	Effect	Mechanism	References
Asthma	Female SD mice	OVA	1, 2 mg/kg, i.p	14 days	↓Inflammatory infiltration, AHR; ↑IL-2, ↓IL-4; ↓TLR4, NF-κB	TLR4/NF-κB	Liu et al. (2023)
Asthma	Pediatric peripheral blood mononuclear cells (PBMCs)		0.1, 0.5, 2 μmol/L	48 h	↓TLR4, MyD88, NF-Κb; ↓TNF-α, IL-6	TLR4/NF-κB	Yu et al. (2019)
Asthma	Human lung airway epithelial cells (H292)	EGF	1, 5 μmol/L	24 h	↓p-AKT; ↓MUC5AC	AKT and NF-κB	Wu et al. (2023)
Asthma	Murine alveolar macrophages (MH-S)	LPS	1, 5 μmol/L	24 h	↓IL-6, NF-κB p-P65	AKT and NF-κB	Wu et al. (2023)
Asthma	Female BALB/c mice	OVA	10 mg/kg, i.p	3 days	↓Mucus overproduction, AHR; ↓IL-4, IL-5, IL-13; ↓GABA_A_R	GABAAR	Shen et al. (2016)
Asthma	Female C57BL/6 mice	OVA/LPS	20 mg/kg, i.p	25 days	↓Inflammatory cells, mucus; ↓Ly6G, IL-36γ; ↓p-p38 MAPK, p-ERK, p-JNK; ↓IL-1β	MAPK	Qiao et al. (2023)
Asthma	Male SD rats	OVA	1 mg/kg, i.p	24 days	↓Neutrophils, Eosinophils; ↓IL-4; ↑PPARγ; ↓p-p38 MAPK	p38 MAPK	Zeng et al. (2014)
Asthma	Human monocytic leukemia cell (THP-1)	PMA (phorbol 12-myristate 13-acetate)	0.1 μmol/L	48 h	↓M1(CD11B, INOS), ↓TNF-α, IL-6; ↑M2 (ARG1, FIZZ1), ↑IL-10, IL-RA; ↑hsa_circ_0001326; ↓hsa-miR-136-5p, ↑USP4	M1/M2 and hsa_circ_0001326	Gong et al. (2022)
Asthma	Male BALB/c mice	OVA	20 mg/kg, i.p	42 days	↓Eosinophils, Neutrophils; ↓IgE, ↓eotaxin2, CCR3; ↓Th2(IL-4, IL-5, IL-13), ↑Th1(IFN-γ); ↑Treg (CD4^+^CD25^+^FOXP3^+^),↑ IL-10, TGF-β1	Th2/Th1 and Treg	Kim et al. (2018)
Asthma	Male BALB/c mice	OVA	10 mg/kg, p.o	6 days	↓AHR; ↓IgE; ↓Th2(IL-4, IL-5); ↑Th1(IFN-γ)	Th2/Th1	Das et al. (2003)
Asthma	Female BALB/c mice	OVA	0.1 mg/kg, i.p	35 days	↓Neutrophils, Eosinophils; ↓Th2(IL-4, IL-5, IL-13)	Th2	Jang et al. (2017)
Asthma	Female BALB/c mice	OVA	10, 20 mg/kg, i.p	7 days	↓Lymphocytes, eosinophils, inflammatory cells; ↓Mucus, collagen, AHR; ↓IgE; ↓Th2(IL-4, IL-5, IL-13); ↓LC3B,↑p62, ↓Beclin-1/PI3KC3; ↓PI3K p85, p-Akt, p-mTOR	Th2 and PI3K/Akt/mTOR	Wang et al. (2021c)
Asthma	Male BALB/c mice	OVA	1 mg/kg, i.g	24 days	↓Airway wall thickness, smooth muscle proliferation; ↓IL-13Rα2, TGF-β1	TGF-β1	Wang et al. (2007)
Asthma	The human bronchial epithelium cells (BEAS-2B)	LPS	20 μmol/L	48 h	↓Th2 (IL-25, 33, TSLP); ↓NF-κB(IκBα, p65); ↓TGF-β1; ↑GSK-3β; ↓β-catenin, ↓α-SMA, FN, MMP-9; ↓N-cadherin, vimentin, Snail; ↑E-cadherin; ↓EMT	EMT	Quan et al. (2024)
Asthma	Male C57BL/6 mice	OVA	10, 20 mg/kg, i.p	7 days	↓Leukocytes, eosinophils; ↓Inflammatory infiltration, mucus; ↓IgE; ↓Th2 (IL-25, 33, TSLP), (IL-13, IL-5, IL-4); ↓NF-κB(IκBα, p65); ↓TGF-β1; ↓β-catenin; ↓α-SMA, FN, MMP-9; ↓N-cadherin, vimentin, Snail; ↑E-cadherin; ↓EMT	EMT	Quan et al. (2024)

↑ = upregulated; ↓ = downregulated.

#### 4.3.1 Inhibition of NF-κB and related inflammatory pathways

In OVA-induced juvenile rat models, luteolin (2 mg/kg, i.p.) markedly reduced inflammatory cell infiltration and AHR by suppressing the TLR4/NF-κB pathway, accompanied by decreased IL-4 and increased IL-2 levels (Liu et al., 2023). A clinical study further confirms that luteolin (2 μmol/L) reduces IL-6 and TNF-α via TLR4/NF-κB signaling, supporting its potential in managing pediatric allergic asthma (Yu et al., 2019). *In vitro*, luteolin (5 μmol/L), an active component of the traditional formulation “Sanzi Yangqin Decoction,” inhibited NF-κB activation and reduced IL-6 levels in LPS-stimulated MH-S macrophages. In H292 airway epithelial cells, luteolin suppressed EGF-induced AKT phosphorylation and downregulated MUC5AC expression, thereby mitigating mucus hypersecretion (Wu et al., 2023). In an earlier OVA-induced guinea pig model, luteolin (30 mg/kg) reduced eosinophil infiltration and bronchial hyperreactivity (Lee et al., 2014).

The γ-aminobutyric acid receptor (GABA_A_R) activity has also been implicated in airway mucus overproduction. In OVA-induced asthmatic mice, luteolin (10 mg/kg, i.p.) was proposed to inhibit GABA_A_R activity, which correlated with decreased goblet cell hyperplasia, mucus secretion, and Th2 cytokines (IL-4, IL-5, and IL-13) in BALF, collectively alleviating airway inflammation (Shen et al., 2016).

#### 4.3.2 Modulation of the MAPK pathway

Luteolin further modulates the MAPK pathway, which is implicated in Th2 inflammation and bronchial remodeling. In OVA-induced murine models, luteolin (20 mg/kg, i.p.) inhibited phosphorylation of p38 MAPK, ERK, and JNK, and reduced Ly6G and IL-36γ expression in lung tissue, and decreased IL-1β levels (Qiao et al., 2023). Similar results were observed in airway epithelial cells, where luteolin (20 μmol/L) suppressed IL-36γ-induced MAPK activation and downstream IL-1β release, indicating its role in mitigating neutrophil-driven asthma by blocking the IL-36γ/MAPK axis (Qiao et al., 2023). Another study in OVA-induced rat models showed that luteolin (1 mg/kg, i.p.) also upregulated PPAR-γ and suppressed p38 MAPK expression, resulting in reduced bronchial wall thickening, inflammatory cell infiltration, and IL-4 levels in BALF (Zeng et al., 2014).

Recent evidence shows that pollenin B, a flavonoid compound isolated from Ephedrae Herba, attenuates airway hyperresponsiveness by activating PPAR-γ and modulating arachidonic acid metabolism and inflammatory signaling (Wu et al., 2025b). This mechanism parallels luteolin’s ability to activate PPAR-γ, underscoring this nuclear receptor as a common regulatory node for flavonoids in inflammatory airway diseases.

#### 4.3.3 Restoration of immune homeostasis and autophagy regulation

Asthmatic inflammation arises from a breakdown in both innate and adaptive immune balance. In THP-1-derived macrophages, luteolin (0.1 μmol/L) shifted macrophage polarization toward the M2 phenotype, as shown by reduced expression of M1 markers (CD11B, iNOS) and inflammatory cytokines (TNF-α, IL-6), alongside increased expression of M2 markers (Arg1, FIZZ1) and anti-inflammatory cytokines (IL-10, IL-1RA). Furthermore, luteolin upregulated hsa_circ_0001326, which was proposed to inhibit the miR-136-5p/USP4 axis, thereby further promoting M2 polarization (Gong et al., 2022).

In OVA-induced murine models, luteolin (10 mg/kg, i.p.) diminished eosinophil and neutrophil counts in BALF, lowered OVA-specific IgE and eotaxin-2/CCR3 levels, and rebalanced Th1/Th2 responses by elevating IFN-γ while reducing IL-4, IL-5, and IL-13 levels (Das et al., 2003; Jang et al., 2017; Kim et al., 2018). The treatment also expanded the CD4^+^CD25^+^Foxp3^+^ Treg population, which was associated with increased levels of IL-10 and TGF-β1, thereby supporting the restoration of immune homeostasis (Kim et al., 2018).

Luteolin further influences autophagic processes. In a murine asthma model, luteolin (20 mg/kg, i.p.) activated the PI3K/Akt/mTOR pathway and inhibited Beclin-1/PI3KC3 complex assembly, leading to inhibiting aberrant autophagy—evidenced by downregulated LC3B and accumulated p62—and concomitant decreases in Th2 cytokines (IL-4, IL-5, IL-13) levels (Wang S. et al., 2021). Through this coordinated regulation of immune responses and autophagy, luteolin mitigates core asthmatic features including AHR, mucus overproduction, and airway remodeling.

#### 4.3.4 Suppression of EMT and airway remodeling

Epithelial–mesenchymal transition (EMT) is a key process in asthmatic airway wall thickening and remodeling, primarily driven by TGF-β1. In OVA-induced murine models, luteolin (1 mg/kg, i.g.) suppressed airway wall thickening and smooth muscle proliferation, an effect associated with downregulation of TGF-β1 and IL-13Rα2 expression (Wang et al., 2007). In LPS-stimulated BEAS-2B cells, luteolin inhibited Th2-associated upstream epithelial cytokines (IL-25, IL-33, TSLP) by blocking NF-κB activation, and also suppressed TGF-β1 expression and β-catenin activation, collectively attenuating EMT progression (Quan et al., 2024).

Further confirming this protective role, luteolin (20 mg/kg, i.p.) reduced inflammatory cell infiltration, mucus production, and serum IgE levels in OVA-induced asthmatic mice. The treatment also downregulated upstream epithelial cytokines (IL-25, IL-33, TSLP) and Th2 inflammatory cytokines (IL-4, IL-5, IL-13), thereby alleviating airway inflammation and remodeling.

Furthermore, mechanistic studies revealed that luteolin inhibited NF-κB signaling and Snail expression, while upregulating GSK-3β to prevent β-catenin activation, leading to suppression of TGF-β1-mediated EMT, as evidenced by reduced fibrotic and mesenchymal markers, including α-SMA, fibronectin (FN), MMP-9, N-cadherin, and vimentin, alongside restored E-cadherin expression, ultimately improving airway structure and function (Quan et al., 2024).

### 4.4 Role of luteolin in COPD

Chronic obstructive pulmonary disease (COPD) is a progressive respiratory disorder driven largely by chronic exposure to cigarette smoke (CS) and airborne pollutants. The disease manifests as persistent neutrophilic inflammation, oxidative stress, and progressive airway remodeling. The potential protective mechanisms of luteolin are summarized in Table 3.

**TABLE 3:** Potential molecular mechanisms of Luteolin in treating COPD and pulmonary fibrosis.

Disease	Type	Model	Dose	Duration	Effect	Mechanism	References
COPD	Male BALB/c mice	CS	40 mg/kg, i.g	75 days	↓MDA, ↑SOD, CAT; ↓IL-1β, IL-6, TNF-α, IL-8; ↑HO-1, NQO-1; ↓NOX4; ↓NF-Κb	NOX4/NF-κB	Li et al. (2023)
COPD	Human alveolar epithelial (A549)	CSE	100 μmol/L	24 h	↓MDA, ↑SOD, CAT; ↓IL-1β, IL-6, TNF-α, IL-8; ↑HO-1, NQO-1; ↓NOX4; ↓NF-κB	NOX4/NF-κB	Li et al. (2023)
COPD	Human		15 mg/kg, p.o	14 days	↓TNF-α; ↑FEV1 (pred%), FEV1/FVC, MVV, PEF	Clinical application	Tang et al. (2012)
COPD	Human		15 mg/kg, p.o	14 days	↓TNF-α, IL-6, IL-12; ↑FEV1 (pred%), FEV1/FVC, MVV	Clinical application	Li (2016)
COPD	Male C57BL/6J mice	CS + LPS	50 mg/kg, i.g	48 days	↑SIRT6, ↓TRPV1, CYP2A13; ↑NRF2; ↑SOD1, SOD2, PGC1α; ↓MDA, LDH, ↑SOD, CAT, GSH	TRPV1 and CYP2A13/NRF2	Zhou et al. (2024)
COPD	Human alveolar epithelial (A549)	CSE	30 μmol/L	12 h	↓ROS; ↓MDA, LDH,↑SOD; ↓Ca^2+^ influx; ↑SIRT6, ↓TRPV1, CYP2A13; ↑NRF2	TRPV1 and CYP2A13/NRF2	Zhou et al. (2024)
Pulmonary Fibrosis	SD rats	Bleomycin	40 mg/kg, i.g	14 days	↓HYP; ↓TGF-β1	TGF-β1	Gong et al. (2005)
Pulmonary Fibrosis	Male C57BL/6 mice	Bleomycin	10 mg/kg, p.o	21 days	↓Inflammatory infiltration; ↓Collagen deposition; ↓TNF-α, IL-6; ↓TGF-β1	TGF-β1	Chen et al. (2010)
Pulmonary Fibrosis	Primary Cultured Lung Fibroblasts, Human alveolar epithelial (A549)	TGF-β1	25 μmol/L	48 h	↑E-cadherin; ↓R-SMA, collagen I, vimentin; ↓EMT; ↓p-Smad2/3	EMT	Chen et al. (2010)
Silicosis Fibrosis	Male Wistar rats	SiO2	20, 40, 80 mg/kg, i.g	28 days	↓ IL-1β, IL-18; ↓TGF-β1; ↓NLRP3, Caspase-1	TGF-β1	Song (2019)
Silicotic Fibrosis	Human normal lung epithelial cells (Beas-2B)	TGF-β1	20 μmol/L	2 h	↑E-cadherin; ↓α-SMA, vimentin, N-cadherin; ↓COL1A1, COL3A1; ↓EMT; ↓BCL-2	EMT	Li et al. (2024b)
Pulmonary Fibrosis	Male C57BL/6 mice	CS	125 mg/kg, p.o	60 days	↓Inflammatory infiltration, Alveolar wall thickening, Airway remodeling; ↑SOD, GSH, CAT; ↓IL-6, TNF-α; ↑IL-10; ↓α-SMA, collagen I, fibronectin, MMP-2/9; ↑E-cadherin; ↓EMT, FMT; ↓p-Smad2/3	EMT, FMT	Peng et al. (2025)

↑ = upregulated; ↓ = downregulated.

#### 4.4.1 Inhibition of the NOX4/NF-κb pathway

NADPH oxidase 4 (NOX4) serves as a major contributor to ROS generation in COPD, promoting oxidative injury and NF-κB-mediated inflammation. Luteolin appears to interfere with this process by modulating the NOX4/NF-κB axis. In cigarette smoke (CS)-exposed murine models, luteolin (40 mg/kg, i.g.) suppressed NOX4 expression, lowered MDA levels, and enhanced antioxidant enzyme activities (SOD, CAT, NQO1, HO-1), and inhibited NF-κB activation, along with decreased levels of IL-1β, IL-6, TNF-α, and IL-8 (Li et al., 2023). Comparable anti-inflammatory outcomes were reproduced in cigarette smoke extract (CSE)-stimulated A549 cells (Li et al., 2023). In another study, luteolin (20 mg/kg), administered as a principal bioactive component of Sanzijing decoction, improved histopathological changes and pulmonary function by downregulating EGFR and MMP9 expression in CS-induced murine models (Wang et al., 2021).Clinical evidence further supports the translational potential of luteolin. Luteolin (15 mg/kg, p.o.) as the main active constituent of *indigo naturalis* extract, significantly reduced serum levels of TNF-α, IL-6, and IL-12 in patients experiencing acute COPD exacerbations after 2 weeks of treatment. These biochemical improvements correlated with better lung function, reflected by increases in forced expiratory volume in 1 s as a percentage of predicted value (FEV1 (pred%)), FEV1/forced vital capacity (FEV1/FVC), maximal voluntary ventilation (MVV), and peak expiratory flow (PEF) (Li, 2016; Tang et al., 2012).

Macrophage M1 polarization and pyroptosis are pivotal in CS-induced COPD. Recent evidence shows that CS-exposed epithelial cells release exosomal lncRNA MEG3, which promotes METTL3-dependent m6A modification of TREM-1 to drive macrophage M1 polarization and pyroptosis (Wang L. et al., 2024). This underscores the importance of epitranscriptomic regulation in shaping macrophage phenotype and provides a novel mechanistic framework for evaluating luteolin’s potential to restore immune homeostasis in COPD.

#### 4.4.2 Activation of NRF2 and inhibition of the TRPV1 pathway

Transient receptor potential vanilloid subtype 1 (TRPV1), a sensor for harmful components in cigarette smoke, triggers Ca^2+^ influx and oxidative stress, thereby aggravating cellular injury. Cytochrome P450 2A13 (CYP2A13) is also involved in the metabolic activation of inhaled toxicants. In a CS/LPS-induced model, luteolin (50 mg/kg, i.g.) activated SIRT6 and inhibited TRPV1 and CYP2A13 expression. These effects were accompanied by upregulation of NRF2 and its target genes (SOD1, SOD2, PGC1α), decreased levels of MDA and LDH, and elevated activities of SOD, CAT, and GSH. Ultimately, these changes helped preserve epithelial integrity and alleviate airway obstruction (Zhou et al., 2024). Consistent results in CSE-stimulated A549 cells show that luteolin (30 μmol/L) reduced mitochondrial ROS, blocked Ca^2+^ influx and TRPV1 signaling, restored SIRT6 and PGC1α function, supporting a role in mitochondrial protection (Zhou et al., 2024).

The SIRT1/FoxO1 pathway is also increasingly recognized as a key regulator of oxidative stress in CS–induced COPD. Recent findings indicated that increased miR-132 suppressed SIRT1, weakened FoxO1-dependent antioxidant defense, whereas miR-132 inhibition restored SIRT1/FoxO1 signaling, improved lung function and reduced apoptosis (Shen et al., 2024). These findings align with the proposed mechanism whereby luteolin alleviates oxidative damage and mitigates COPD through SIRT1-driven signaling networks.

### 4.5 Role of luteolin in pulmonary fibrosis

**FIGURE 4:**
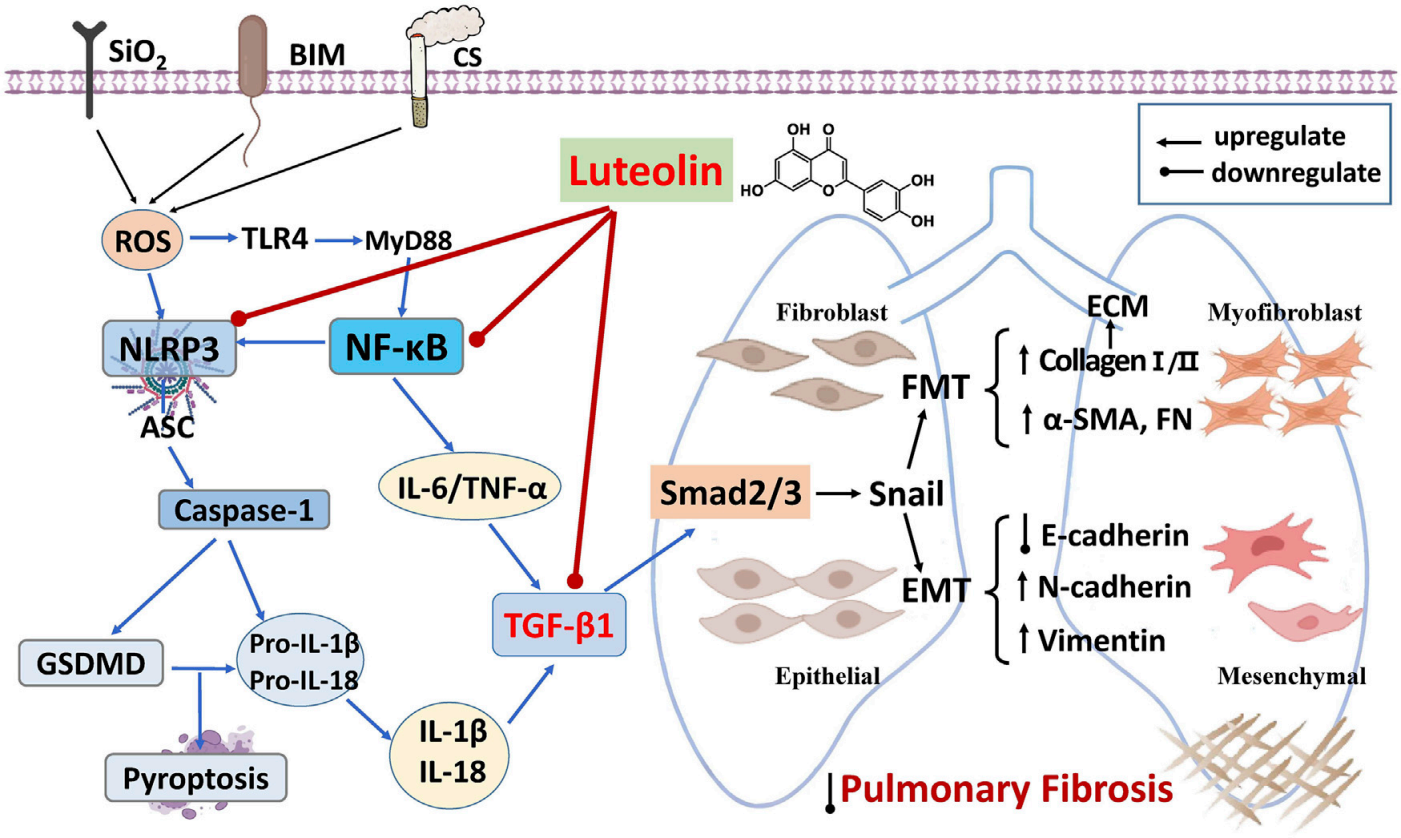
Network regulatory mechanisms of luteolin in treating pulmonary fibrosis.

Pulmonary fibrosis arises from a complex cascade of chronic inflammation, persistent TGF-β1 activation, and aberrant tissue remodeling, which promotes both epithelial-mesenchymal transition (EMT) and fibroblast-to-myofibroblast transition (FMT). These processes are marked by loss of E-cadherin and increased mesenchymal and extracellular matrix (ECM) components such as α-SMA, vimentin, and collagen I, which collectively contribute to fibrotic progression. The potential protective mechanisms of luteolin are illustrated in Figure 4 and summarized in Table 3.

#### 4.5.1 Suppression of pro-fibrotic inflammation

Luteolin counteracts fibrogenesis in part through modulating upstream inflammatory signals that feed into TGF-β1 activation. In bleomycin-induced models, luteolin (40 mg/kg, i.g.) reduced MDA and hydroxyproline (HYP) levels and lowered TGF-β1 mRNA expression (Gong et al., 2005). Similarly, luteolin (10 mg/kg, p.o.) decreased neutrophil infiltration and reduced TNF-α and IL-6 levels in BALF, inhibited TGF-β1 expression and collagen deposition, and ameliorated pulmonary fibrosis (Chen et al., 2010; Pan et al., 2024). In SiO_2_-induced murine silicosis models, luteolin (80 mg/kg, i.g.) interfered with NLRP3 inflammasome assembly and caspase-1 activation, leading to decreased IL-1β and IL-18 release. This attenuated TGF-β1 expression along the NLRP3/TGF-β1/Smad axis and alleviated fibrotic remodeling (Song, 2019).

#### 4.5.2 Direct interference with TGF-β1-driven EMT and FMT

Beyond its anti-inflammatory role, luteolin also directly inhibits TGF-β1- -triggered phenotypic transitions. In human bronchial epithelial Beas-2B cells, luteolin (20 μmol/L) bound B-cell lymphoma 2 (BCL-2), promoted apoptosis, restored E-cadherin expression, and reduced mesenchymal markers (N-cadherin, vimentin) and fibrotic markers (α-SMA and collagen I/III) expression, thereby attenuating TGF-β1-induced EMT and FMT (Li Y. Y. et al., 2024). In murine lung fibroblasts and human A549 cells, luteolin (25 μmol/L) inhibited Smad2/3 phosphorylation and reversed EMT hallmarks by elevating epithelial markers (E-cadherin) and reducing mesenchymal markers (α-SMA, collagen I, vimentin), and exerted potent anti-fibrotic effects (Chen et al., 2010; Han et al., 2024).

Further supporting its therapeutic potential, an extract of *Aconitum flavum*—rich in the luteolin derivative luteolin-7-O-glucuronide (LG)—was shown to ameliorate CS-induced pulmonary fibrosis in mice. The extract reduced inflammatory cell infiltration, attenuated alveolar structural distortion and collagen accumulation, accompanied by decreased TNF-α and IL-6 levels and increased IL-10 expression. Mechanistically, LG directly binds TGF-β1, inhibiting both Smad and non-Smad signaling cascades, upregulates E-cadherin, and downregulates pro-fibrotic mediators (α-SMA, collagen I, fibronectin, MMP-2/9), ultimately blocking TGF-β1-induced EMT and FMT and slowing fibrotic progression (Peng et al., 2025).

## 5 Physicochemical properties and pharmacokinetic profile of luteolin

Luteolin (C_15_H_10_O_6_) is a naturally occurring flavonoid characterized by a polyphenolic structure comprising two aromatic rings (A and B) linked by a central heterocyclic pyran ring (C ring). Its structure contains four hydroxyl groups at positions 5, 7, 3′, and 4′, along with a C_2_ = C_3_ double bond, which together confer both antioxidant potential and a planar backbone. These structural features contribute to moderate lipophilicity (LogP ≈2.5) and significant hydrogen-bonding capacity, though they also lead to poor aqueous solubility (∼10 μg/mL), resulting in an oral bioavailability generally below 20% (Deng et al., 2017; Hayasaka et al., 2018). This poor solubility not only limits intestinal absorption but also constrains systemic distribution, particularly to hydrophilic tissues. To overcome these challenges, researchers have turned to formulation strategies such as nanocarriers, phospholipid complexes, and inhalable formulations, which aim to improve bioavailability and facilitate targeted pulmonary delivery (Chen et al., 2022).

Following oral administration, luteolin is absorbed in its aglycone form and reaches peak plasma concentration within 1–2 h. However, systemic levels of the unmetabolized parent compound remain low due to extensive hepatic metabolism (Deng et al., 2017). Hepatic metabolism of luteolin is primarily mediated by cytochrome P450 enzymes, especially CYP1A1 and CYP3A4. Notably, the expression of these enzymes in pulmonary tissue is substantially lower (10%–20%) than in the liver, contributing to slower metabolic clearance and a prolonged half-life in lung tissue (Hayasaka et al., 2018; Wang et al., 2017). *In vivo* biodistribution studies have demonstrated that, after oral administration (200 mg/kg), luteolin accumulates in lung tissue at levels 3–5 times higher than in plasma, pointing to a preferential pulmonary distribution (Deng et al., 2017). This tissue selectivity is likely attributed to the lung’s dense capillary network and lipophilic microenvironment, which favor the uptake of hydrophobic molecules like luteolin. Consistently, intranasal delivery of luteolin-loaded nanoemulsions has been shown to achieve higher drug levels in the lungs and enhance anti-inflammatory efficacy in murine models (Pan et al., 2024).

Luteolin is eliminated mainly as glucuronide conjugates, with only a small fraction excreted in urine (∼6.6%) and feces (∼31.3%) in its free form, while approximately 70% of its metabolites are cleared renally within 48 h (Hayasaka et al., 2018; Wang et al., 2017). Although these conjugated forms exhibit reduced anti-inflammatory and antioxidant activity, they also display minimal cytotoxicity, which may lower the risk of systemic toxicity during long-term administration (Boersma et al., 2002; Michels et al., 2005). Importantly, the enzyme β-glucuronidase—often enriched at inflammatory sites—can locally hydrolyze these glucuronides to release the active aglycone, enabling targeted drug reactivation precisely where inflammation occurs (Chen et al., 2022).

With its low molecular weight (286.24 Da), hydrophobic nature, and moderate LogP value, luteolin displays favorable membrane permeability, allowing it to passively diffuse into pulmonary epithelial cells and alveolar macrophages. The compound preferentially accumulates in the lipid-rich pulmonary microenvironment, enabling lung-targeted distribution even after systemic administration and thereby reducing exposure to non-target organs (Manach et al., 2005; Kure et al., 2016). Moreover, volume of distribution studies further confirmed its broad tissue penetration, supporting its potential suitability for both systemic and local respiratory treatment (Hayasaka et al., 2018).

Luteolin’s physicochemical properties, including limited solubility, specific metabolic behavior, and preferential distribution within pulmonary tissues, suggest a pharmacological basis for its use in respiratory diseases associated with oxidative stress and localized inflammation.

## 6 Therapeutic strategies of luteolin

### 6.1 Advanced delivery systems for improved targeting

The clinical translation of luteolin faces challenges due to its poor aqueous solubility and low bioavailability. To address this, several nanotechnology-based delivery platforms have been developed to enhance its pulmonary accumulation and therapeutic performance. In murine models of bacterial lung infection, MPEG-PLA micelles loaded with luteolin (Luteolin/MPEG-PLA) significantly improved pulmonary accumulation and anti-inflammatory effects (Miao et al., 2021). Similarly, an inhalable γ-cyclodextrin–metal-organic framework (CD-MOF) nanocarrier facilitated targeted pulmonary delivery, where it suppressed NF-κB and alleviated extracorporeal circulation (ECC)-induced ALI (Ling et al., 2023). For pulmonary fibrosis, dry powder inhalers containing luteolin (LUT@CDMOF) downregulated TGF-β1/Smad3 and attenuated bleomycin-induced interstitial lesions (Ren et al., 2023). Another strategy employed a hyaluronidase-responsive system (Lut@HAase), which reduced markers of fibrosis such as α-SMA and fibronectin, decreased collagen deposition, and improved lung function in murine models (Pan et al., 2024). Additionally, chitosan/sodium alginate hydrogel nanoparticles (NPs@LUT) have also been explored for their ability to enable sustained release of drugs in inflammatory microenvironments and offer antibacterial and anti-inflammatory activity with favorable biocompatibility (Wang J. J. et al., 2025). These advanced systems collectively improve the bioavailability, lung-specific targeting, and pharmacologic profile of luteolin.

### 6.2 Multi-target actions in herbal formulations

Luteolin frequently appears as a key component in traditional multi-herbal formulations, exerting therapeutic effects through complex regulatory networks. Network pharmacology and molecular docking studies suggest that its key targets include EGFR, MMP9, PTGS2, and APP, with involvement in pathways such as PI3K-Akt, HIF-1α, and NF-κB. For example, in “Sanzi Yangqin” decoction, luteolin inhibits EGF-induced AKT activation and MUC5AC expression, thereby improving asthmatic symptoms (Wu et al., 2023). In COPD models, it helps restore lung function by regulating targets including EGFR, MMP2/9, APP, and ERBB2 (Wang et al., 2021). In “Shenqi Tiaoshen” and “Modified Bushen Yiqi” decoctions, luteolin appears to modulate MMP9, NF-κB, AP-1, and PI3K-Akt/HIF-1α pathways, reducing cytokine expression and improving pulmonary function (Choi et al., 2024; Yang et al., 2024). Furthermore, in “Wenfei Fuyang Qutan,” luteolin synergizes with kaempferol and quercetin to regulate PI3K-Akt, NCOA2, and PTGS2, forming an integrated multi-target network (Feng et al., 2024).

### 6.3 Combination therapy and mechanistic synergy

Luteolin’s therapeutic efficacy can be potentiated through combination with other bioactive agents. In a murine ALI model, the co-administration of luteolin (15 mg/kg) with paeoniflorin (75 mg/kg) synergistically inhibited NF-κB and MAPK signaling, leading to stronger suppression of inflammation and oxidative stress (Liu Z. et al., 2024). In fibrotic models, luteolin (10 μmol/L) combined with kaempferol (5 μmol/L) activated the AMPK/PPAR-γ signaling and inhibited TGF-β1-induced α-SMA expression, thereby blocking FMT and ECM accumulation (Yan et al., 2023). These combinatorial approaches enhance the therapeutic potential of luteolin in the management of diverse pulmonary diseases.

## 7 Conclusion

Luteolin, a naturally occurring flavonoid, demonstrates a favorable safety profile and notable pulmonary selectivity, alongside its multi-target pharmacological activities against oxidative stress, inflammatory signaling, and immune dysregulation. These properties support its therapeutic potential across a spectrum of acute and chronic inflammatory respiratory diseases. The core mechanisms include:

1. Suppression of pro-inflammatory and oxidative signaling: Luteolin directly inhibits key pathways such as NF-κB, AP-1, and MAPK (p38, ERK, JNK), while modulating oxidative stress-related axes including Nrf2/HO-1 and HIF-1α/NLRP3. These actions collectively reduce the release of pro-inflammatory cytokines (e.g., TNF-α, IL-6) and attenuate ROS-mediated damage.
2. Regulation of innate immune activation: Luteolin inhibits the TLR4/NF-κB axis, NLRP3 inflammasome assembly, and both caspase-1– and caspase-11-dependent pyroptosis, thereby curbing excessive innate immune responses.
3. Restoration of adaptive immune homeostasis: Luteolin restores immune balance by regulating macrophage M1/M2 polarization, rebalancing Th1/Th2 differentiation, enhancing Treg cell function, and bidirectionally modulating TGF-β1 signaling, supporting resolution of inflammation and restoration of lung tissue injury.
4. Attenuation of fibrotic remodeling: Luteolin suppresses TGF-β1-driven EMT and FMT, reducing collagen deposition and pathological airway remodeling in chronic respiratory conditions.

Despite these advantages, several challenges require attention. First, the systems-level integration of luteolin’s multi-target actions remains incompletely understood. Future studies should incorporate transcriptomic, proteomic, and metabolomic approaches to clarify its network-based regulatory effects. Second, its poor oral bioavailability and metabolic instability highlight the need for advanced delivery systems—such as nanoparticle formulations, liposomes, or inhalable preparations—to enhance its pharmacokinetic profile. Third, robust clinical evidence remains limited, and well-designed randomized controlled trials will be essential to confirm its efficacy and safety in human populations.

In summary, luteolin represents a promising multi-target candidate for respiratory diseases, owing to its favorable safety profile and substantial potential. Further research should prioritize mechanistic elucidation, delivery optimization, and clinical translation to support its development into a viable therapeutic option.

## Glossary

AHR: Airway hyperresponsiveness
AKT: Protein Kinase B
ALI: Acute Lung Injury
AP-1: Activator Protein-1
Arg1: Arginase 1
ASC: Apoptosis-Associated Speck-Like Protein Containing a CARD
BALF: Bronchoalveolar Lavage Fluid
cAMP: Cyclic Adenosine Monophosphate
Caspase-1: Cysteinyl aspartate specific proteinase-1
cGMP: Cyclic Guanosine Monophosphate
CIRP: Cold-Inducible RNA-Binding Protein
CLP: Cecal Ligation and Puncture
COPD: Chronic Obstructive Pulmonary Disease
CS: Cigarette smoke
CSE: Cigarette Smoke Extract
CSI: Cecal slurry injection
CYP2A13: Cytochrome P450 Family 2 Subfamily A Member 13
CYP450: Cytochrome P450
ECC: Extracorporeal Circulation
EGF: Epidermal Growth Factor
EGFR: Epidermal Growth Factor Receptor
EGR1: Early Growth Response 1
EMT: Epithelial-to-Mesenchymal Transition
ENaC: Epithelial Sodium Channel
ERK: Extracellular Signal-Regulated Kinase
fMLP: formyl-Met-Leu-Phe
FMT: Fibroblast-to-Myofibroblast Transition
Foxp3: Forkhead Box P3
GABAAR: Gamma-Aminobutyric Acid A Receptor
GPx: Glutathione Peroxidase
GSDMD: Gasdermin D
HIF-1α: Hypoxia-Inducible Factor 1 Alpha
HO-1: Heme Oxygenase-1
HPH: Hypoxic pulmonary hypertension
HYP: Hydroxyproline
ICAM-1: Intercellular Adhesion Molecule-1
IFN-β: Interferon Beta
IgE: Immunoglobulin E
IκBα: Inhibitor of Nuclear Factor Kappa B Alpha
JAK2: Janus Kinase 2
JNK: c-Jun N-Terminal Kinase
KC: Keratinocyte-Derived Cytokine
MAPK: Mitogen-Activated Protein Kinase
MEK: MAPK/Extracellular Signal-Regulated Kinase
MIP-2: Macrophage Inflammatory Protein-2
MMP: Matrix Metalloproteinase (e.g. MMP-2, MMP-9)
MPO: Myeloperoxidase
mTOR: Mammalian Target of Rapamycin
NLRP3: NOD-Like Receptor Pyrin Domain–Containing 3
NOS2: Nitric Oxide Synthase 2
NOX4: NADPH Oxidase 4
NQO1: NAD (P)H Quinone Dehydrogenase 1
Nrf2: Nuclear Factor Erythroid 2-Related Factor 2
OVA: Ovalbumin
P65: NF-κB p65 Subunit
PDE4: Phosphodiesterase 4
PI3K: Phosphoinositide 3-Kinase
PI3KC3: Class III Phosphatidylinositol 3-Kinase
PPARγ: Peroxisome Proliferator–Activated Receptor Gamma
RSV: Respiratory Syncytial Virus
sE-selectin: Soluble E-Selectin
sICAM-1: Soluble Intercellular Adhesion Molecule-1
SIRT6: Sirtuin 6
Smad: Small Mothers Against Decapentaplegic
Snail: Snail Family Transcriptional Repressor
SOCS3: Suppressor of Cytokine Signaling 3
STAT3: Signal Transducer and Activator of Transcription 3
TGF-β1: Transforming Growth Factor Beta 1
Th1/Th2: T Helper Cell Type 1/Type 2
TLR4: Toll-Like Receptor 4
Treg: Regulatory T Cells
TRPV1: Transient Receptor Potential Vanilloid 1
TXNIP: Thioredoxin-Interacting Protein
VCAM-1: Vascular Cell Adhesion Molecule-1
α-SMA: Alpha-Smooth Muscle Actin
